# Juvenile Spongiotic Gingival Hyperplasia: Clinicopathological Features of Eight Cases and a Literature Review

**DOI:** 10.3290/j.ohpd.a45435

**Published:** 2020-10-27

**Authors:** Lara Maria Alencar Ramos Innocentini, Thais Tedeschi dos Santos, Marília Bianchini Lemos Reis, Carolina de Moraes Rego Mandetta, Rafael Moraes Chaves Santos, Ricardo Alves Mesquita, Tarcília Aparecida da Silva, Soraya de Mattos Camargo Grossmann, Celso Augusto Lemos, Márcio Fernando de Moraes Grisi, Jorge Esquiche León, Ana Carolina Fragoso Motta

**Affiliations:** a Dentist, Department of Stomatology, Public Oral Health and Forensic Dentistry, School of Dentistry of Ribeirão Preto, University of São Paulo, Ribeirão Preto, Brazil; Dentistry and Stomatology Division, Ophthalmology, Otolaryngology and Head and Neck Surgery Department, Ribeirão Preto Clinical Hospital, School of Medicine, São Paulo University, Ribeirão Preto, Brazil. Drafted and wrote the manuscript, contributed to analysis of data and critical review of the manuscript.; b Dentist,Department of Stomatology, Public Oral Health and Forensic Dentistry, School of Dentistry of Ribeirão Preto, University of São Paulo, Ribeirão Preto, Brazil. Contributed to clinical data, drafted the manuscript, analysis of data, critical review of the manuscript.; c Dentist, Department of Oral and Maxillofacial Surgery, and Periodontology, School of Dentistry of Ribeirão Preto, University of São Paulo, Ribeirão Preto, SP, Brazil. Contributed to clinical data, drafted the manuscript, contributed to analysis of data and critical review of the manuscript.; d Dentist, Department of Oral and Maxillofacial Surgery, and Periodontology, School of Dentistry of Ribeirão Preto, University of São Paulo, Ribeirão Preto, SP, Brazil. Data analyses, literature review, drafted the manuscript, contributed substantially to discussion, analysis of data and critical review of the manuscript.; e Dentist, Department of Oral and Maxillofacial Surgery, and Periodontology, School of Dentistry of Ribeirão Preto, University of São Paulo, Ribeirão Preto, SP, Brazil. Contributed to clinical data, drafted the manuscript, contributed to analysis of data and critical review of the manuscript.; f Professor, Department of Oral Pathology and Surgery, Federal University of Minas Gerais, Belo Horizonte, Brazil. Data analyses, literature review, drafted the manuscript, contributed substantially to discussion, contributed to analysis of data and critical review of the manuscript.; g Professor, Department of Oral Pathology and Surgery, Federal University of Minas Gerais, Belo Horizonte, Brazil. Data analyses, literature review, drafted the manuscript, contributed substantially to discussion, analysis of data, critical review of the manuscript.; h Professor, Department of Dentistry, Pontifical Catholic University of Minas Gerais, Belo Horizonte, Brazil. Data analyses, literature review, drafted the manuscript, contributed substantially to discussion, contributed to analysis of data and critical review of the manuscript.; i Professor, Department of Stomatology, School of Dentistry, University of São Paulo, São Paulo, Brazil. Data analyses, literature review, drafted the manuscript, contributed substantially to discussion, contributed to analysis of data and critical review of the manuscript.; j Professor, Department of Oral and Maxillofacial Surgery, and Periodontology, School of Dentistry of Ribeirão Preto, University of São Paulo, Ribeirão Preto, SP, Brazil. Contributed to clinical data, drafted the manuscript, contributed to analysis of data and critical review of the manuscript.; k Professor, Department of Stomatology, Public Oral Health and Forensic Dentistry, School of Dentistry of Ribeirão Preto, University of São Paulo, Ribeirão Preto, Brazil. Contributed to clinical data, drafted the manuscript, contributed to analysis of data and critical review of the manuscript.; l Professor, Department of Stomatology, Public Oral Health and Forensic Dentistry, School of Dentistry of Ribeirão Preto, University of São Paulo, Ribeirão Preto, Brazil. Data analyses, literature review, drafted the manuscript, contributed substantially to discussion and critical review of the manuscript.

**Keywords:** gingival diseases, hyperplasia, inflammatory gingival hyperplasia, junctional epithelium, spongiotic gingival hyperplasia

## Abstract

**Background:**

Juvenile spongiotic gingival hyperplasia (JSGH) is a benign proliferation of non-keratinised stratified squamous epithelium with evident spongiosis, exocytosis leukocytes and dilated vessels with varying numbers of inflammatory cells. Although uncertain, it has been suggested that the epithelial proliferation is probably related to trauma and local irritants. It emerges as a painless erythematous patch or papule with an irregular surface.

**Case reports:**

We present a series of eight cases and a literature review of the clinical presentation and treatment approaches for JSGH. This series includes four females and four males with a mean age of 11.6 years, all complaining of injury to the gums. All lesions were painless and not bleeding. One patient associated the lesion with the onset of trauma and another with a previous history of an eruption cyst. Periapical radiographs of the adjacent area did not show any alteration of bone or teeth. Based on these findings, the diagnostic hypotheses were a non-neoplastic proliferative process or a factitious injury. Incisional biopsy was performed and histopathologic diagnosis was conclusive for JSGH. Surgical excision was conducted in three cases, and the other five cases were supported by clinical observation and plaque control in order to prevent gingival recession defects. After a follow-up period of 11 months (mean), no recurrence was observed for surgically treated cases and mild improvement was achieved with periodontal treatment.

**Conclusion:**

This case series illustrates the importance of diagnosis and follow-up of patients with JSGH. A conservative approach consisting of basic periodontal support instead of surgical excision may be indicated because of the risk of aesthetic defects at the involved areas.

Juvenile spongiotic gingival hyperplasia (JSGH) is an inflammatory hyperplasia with a singular clinicopathologic characteristic and unknown aetiology, which affects the gingiva of children and young individuals.^4^ The lesion was first described by Darling et al^7^ as juvenile spongiotic gingivitis from a clinical, histopathological and immunohistochemical investigation of 24 cases,^7^ and subsequently defined as localised JSGH by Chang et al^4^ based on the variation of the clinical and histopathological presentation of a larger population.^4^

Clinically, JSGH mostly occurs in the attached gingiva, not necessarily affecting marginal gingiva, and does not appear to be related to dental biofilm,^4^,^7^ though the presence of plaque may worsen this condition.^17^ Lesions can be focal or multifocal, and present as bright red raised overgrowths, painless, and most often with a subtle papillary or fine granular surface.^4^ Histopathologic features are unique and characterised by non-keratinised epithelial hyperplasia with exuberant spongiosis and neutrophilic exocytosis.^4^,^7^ The aim of this case series is to describe the clinical and histopathological characteristics of eight patients with JSGH. The differential diagnoses, treatment and follow-up of this condition were also discussed in light of a review of previously reported cases.

## Case Reports

**Fig 1:**
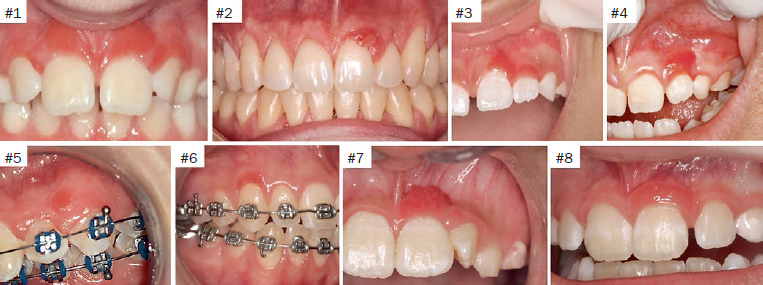
Clinical features of eight cases of JSGH: painless lesion, bright red coloration, well demarcated, slightly raised, with a subtle papillary architecture.

**Fig 2:**
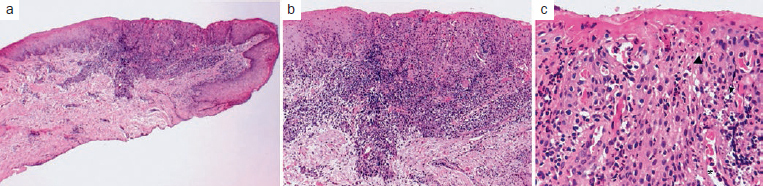
(a) Histopathological characterisation with photomicrographs stained with haematoxylin and eosin showing benign proliferation of non-keratinised stratified squamous epithelium; (b) connective tissue shows a moderate inflammatory infiltrate consisting predominantly of lymphocytes and plasma cells; (c) evident spongiosis in the epithelium (arrow), leukocyte exocytosis (arrow head) and dilated vessels (asterisk) with variable numbers of plasma cells.

**Fig 3:**
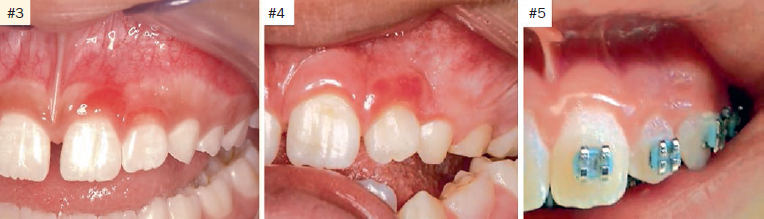
Follow-up of three cases (3, 4, and 5).

Demographic data, clinical information, treatment and the outcome of eight patients with JSGH were reviewed and summarised in Table 1. Patients were seen between 2013 and 2018, and at the time of diagnosis, the age of the patients ranged from 6 to 24 years with a mean of 11.6 years. Four patients were female and four were male, one patient was black and seven were white; all were students. The clinical features of all cases are illustrated in Figs 1 and 3. All cases showed red papules or patches on the vestibular gingiva of the anterior region of maxilla, only cases 1 and 3 presented more than one site compromised by lesions. The size of the lesions ranged from 0.3 to 1.0 cm. Most of the cases affected either the marginal or attached gingiva, except for case 5, which occurred exclusively on the attached gingiva as a localised single lesion (Fig 1). The duration of the lesions ranged from 2–15 months (mean 6.2 months). Histopathological features were characterised by benign proliferation of non-keratinised stratified squamous epithelium, with evident spongiosis, leukocyte exocytosis and dilated vessels with variable numbers of plasma cells from lesion biopsies (Fig 2). Surgical excision was performed in three cases (2, 5 and 6), and the other five cases were supported by clinical observation in order to prevent gingival recession defects. Complete surgical removal was not proposed for those cases with risk of causing gingival recession, as shown in the case 3. In addition, a oral hygiene instructions were given to all patients. The follow-up period ranged from 4 to 15 months. No recurrence was observed in the cases 5 and 6, and the lesions persisted in the other five cases, but showed mild improvement compared to the clinical features at the first appointment (Fig 3).

**Table 1:** Demographic and clinical data of eight patients with juvenile spongiotic gingival hyperplasia (JSGH)

Case	Age (years)	Sex	Race	Occupation	Time of disease (months)	Site	Treatment	Recurrence
1	10	M	W	Student	3	Marginal and attached gingiva	Dental biofilm control	No
2	10	F	W	Student	8	Marginal and attached gingiva	Dental biofilm control	Yes
3	6	F	W	Student	15	Marginal and attached gingiva	Dental biofilm control	Yes
4	14	F	W	Student	2	Marginal gingiva	Surgical excision	No
5	11	F	W	Student	3	Attached gingiva	Surgical excision	No
6	24	M	W	Student	6	Marginal and attached gingiva	Surgical excision	No
7	9	M	B	Student	3	Marginal and attached gingiva	Dental biofilm control	Yes
8	9	M	W	Student	24	Marginal and attached gingiva	Dental biofilm control	Yes

F: female; M: male; B: black; W: white.

## Discussion

JSGH is an uncommon lesion with 193 cases reported in the English-language literature (Table 2). Recently, Vargo and Bilodeau^21^ presented 28 cases with 5 cases occurring in adults, and suggested a review of nomenclature ‘juvenile’ and ‘localised’. Indeed, three of our cases were non-localised and one case was not in a child.

**Table 2:** Clinical-demographic features of cases reported in the English-language literature

Author/year	Number	Male/female	Age	IHC study	Treatment	Recurrence
Darling et al, 2007^7^	24	12/12	5-28	Ck5/6 (+); CK19 (+); estrogen and progesterone (-)	Surgical (23) None (1)	20%
Chang et al, 2008^4^	52	16/36	7-39	None	Surgical (52)	6%
Damm, 2009^6^	1	0/1	10	None	None	No
MacNeill et al, 2011^13^	1	1/0	14	None	Laser CO_2_ cauterization + 0.05% clobetasol ointment	1
Decani et al, 2013^9^	1	0/1	11	None	WD	WD
Solomon et al, 2013^19^	3	2/1	9-15	None	Surgical	2
Petrutiu et al, 2014^16^	3	½	13-15	None	Surgical (2) Surgical + plaque control (1)	0
de Freitas et al, 2015^8^	1	1/0	18	None	None	–
Argyris et al, 2015^2^	21	14/7	8-36	p16INK4A (+)	Surgery (21)	2 (9.5%)
Allon et al, 2016^1^	10	5/5	9-16	CK1/10, CK4, CK8/18, and CK19	WD	WD
Delli et al, 2017^10^	1	0/1	49	None	Surgery	1
Kalogirou et al, 2017^12^	2	2/0	12	CK 19 (+); CK18 (+)	Surgery	0
Nogueira et al, 2017^14^	2	0/2	9-11	None	Cryotherapy	0
Fernandes et al, 2017^11^	4	2/2	9-17	None	Surgery and topical steroids	1
Vieira et al, 2018^21^	1	1/0	9	None	Photodynamic therapy	0
Siamantas et al, 2018 18	1	0/1	19	CK19	Surgery	1
Vargo and Bilodeau, 2018^20^	28	15/13	3-64	CK19	Surgery	3
Lafuente-Ibáñez Mendoza et al, 2019^23^	10	-	9-17	CK19, CK14, Ck7, Ki-67, CD3, CD20, S100	–	–
Wang and Jordan, 2019^13^	27	17/10	≤10-72	CK19 (+); estrogen and progesterone (-)	–	–

WD: without data.

**Fig 4:**
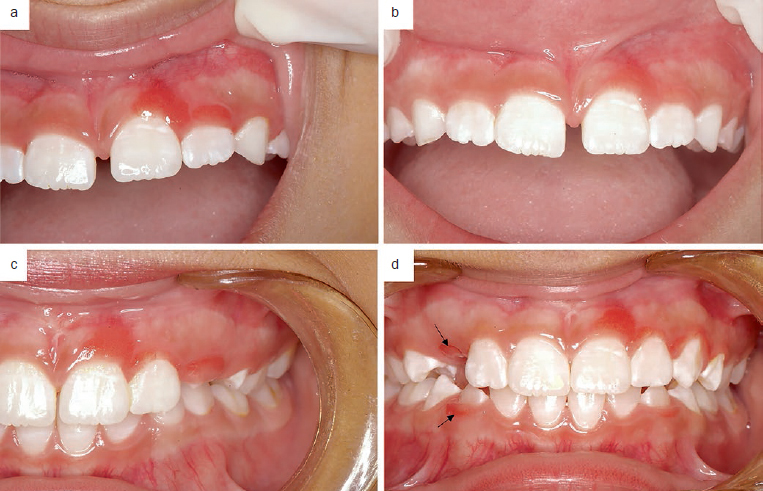
Clinical features of patient 3. (a) Initial appearance of lesion; (b) clinical presentation showing an improvement of lesion with complete regression in left lateral maxilar incisor; (c) two-year follow-up showing other lesions at gingival margin of exfoliated tooth; (d) three-year outcome showing new lesions in mandibular and maxillary buccal gingiva in recently erupted teeth (arrows).

Histologically, JSGH is characterised by benign proliferation of non-keratinised stratified squamous epithelium, with evident spongiosis, neutrophil exocytosis and dilated vessels with variable numbers of lymphocytes and plasma cells.^7^ The nature of this epithelial proliferation was first hypothesised by Darling et al^7^ to originate from junctional epithelium with CK 19 immunohistochemistry expression, and recently by CD117 immunostaining.^1^ Additionally, the authors suggested that the phenotypic profile of junctional epithelium occurs because the gingival sulcus could exteriorise and generate the lesion.^1^ Recently, Lafuente-Ibáñez Mendoza et al^23^ suggested JSGH as an entity of odontogenic origin, in which junctional epithelium probably came from this residual tissue retained in the gingiva after exfoliation of deciduous teeth. In case 3 of this series, we observed the presence of lesions during tooth eruption and in a long-term follow-up (Fig 4).

The main differential diagnosis should include puberty-associated gingivitis,^5^ since the location of the lesion, the affected age and the clinical characteristics may be similar, but this condition usually comprises interproximal areas and is generalised on marginal gingiva. Otherwise, this condition responds to oral hygiene, and with immunostaining, gingival tissue shows positive for estrogen and progesterone receptors.^18^,^23^ In addition, plasma cell gingivitis, inflammatory lesions including pyogenic granuloma as a reactive process, gingival hyperplasia, manifestation of autoimmune diseases and linear gingival erythema should be differentiated from JSGH^16^,^19^,^20^ and squamous papilloma in children.^3^ Among all these differential diagnoses, plasma cell gingivitis is the most similar lesion in clinical appearance but different in histopathology.^18^ In these cases, if no allergen is identified, histopathological examination can show dense plasma cell infiltrate in sub-epithelial connective tissue, which rules out JSGH. Our case series showed no identifiable foreign body associated with the onset of lesions, and histological features were not compatible.^18^ Nevertheless, final diagnosis may be performed with clinical features, response to hygiene protocols and, if possible, with biopsy.

The treatment of JSGH is difficult, and there are still no guidelines with randomised clinical trials.^1^,^8^ However, some authors^17^ have shown improvement with dental biofilm control, as demonstrated by one of our cases (3) (Fig 4). Considering aesthetic aspects, complete surgical excision is chosen when no recession is expected or when gingival plastic surgery is possible. In some cases, follow-up and basic periodontal support is recommended to control lesion progression and relapse of treated lesions (Table 2). Recently, Nogueira et al^15^ reported two successfully treated cases with cryotherapy and proposed this technique for the treatment of JSGH, especially in paediatric patients, since it has some advantages such as ease of application, only minor discomfort, absence of bleeding and satisfactory aesthetic outcomes.^15^ Another conservative approach recently described is photodynamic therapy which showed partial response in one case and considered bacterial plaque control as an important factor in the control of disease.^22^ Indeed, oral hygiene orientation and plaque control are important, because these lesions may promote dental plaque retention and increase gingival inflammation. Additional inflammation caused by dental plaque could may bleeding and biting or chewing impairment. Moreover, the decision between surgical and non-surgical approaches is related to the number of areas involved and the presence of irritants; in some cases, there may be a good indication for conservative and alternative therapies, given the non-aggressive nature of JSGH.

## Conclusion

This case series emphasises clinical aspects of JSGH, in order to differentiate it from other hyperplastic or inflammatory processes of similar clinical characteristics, and illustrates the relevance of correct diagnosis of cases presented in order to establish an adequate treatment plan. Follow-up with basic periodontal support and a more conservative approach are very important especially in aesthetic areas.
